# Foreign Correspondence

**Published:** 1843-02-04

**Authors:** 


### FOREIGN CORRESPONDENCE.

PARIS, Dec. 15, 1842.

*Opening of the Faculty of Medicine.—Prof. Trousseau's Address.—Large doses of the Sulphate of Quinine in Rheumatism.—Its Contra-Stimulant Action.—Claims of Razori to this Treatment.—Serious accidents in five cases from this treatment, and two deaths. The Discussion on Tenotomy at the Academy of Medicine.—MM. Guérin, Bouvier, and Velpeau.*

To the Editor of the Medical Examiner.

Sir,—The School of Medicine opened its annual course, as usual, by public session, for the distribution of the prizes awarded during the vacation. It was generally understood that the opening address would be delivered by Dr. Trousseau. The great reputation of this young and handsome professor as an orator excited general curiosity, and at an early hour the great hall was quite filled. The subject was a parallel between the numerical method and that by induction. It was indeed a brilliant and spirited attack on statistics, which he accused of retarding the progress of science, of fettering the mind, and of reducing the physician to play the character of a mere mechanical counter, without any exercise of intellect on his part. It was better, he said, to advance in darkness, than to stand still. His whole address was listened to with the greatest attention and enthusiasm, and was loudly and frequently applauded. Its diction was elegant and choice; its delivery easy and graceful; and its philosophic character was relieved by agreeable and racy anecdotes.

Dr. Briquet lately communicated to the Academy of Medicine an interesting paper on the employment of sulphate of quinine in large doses in acute rheumatism, in the inflammatory stage. Dr. Rognetta, an Italian oculist, of Paris, has since addressed a letter to the Academy, in which he claims the priority of this practice for Rasori, who, proved, he says, long ago, that the true action of quinine and its preparations was contra-stimulant, and he was hence led to prescribe it in a number of acute and chronic inflammations, and, amongst others, in acute rheumatism. Dr. Mojon, of Genoa, employed the sulphate of quinine in an epidemic of acute rheumatism in 1829, and with the greatest success. Dr. Giacomini, in his Treatise on Therapeutics, states, that from experiments made upon himself, upon patients, and upon animals, he has ascertained that the sulphate of quinine has a very decided hyposthenic action upon the arterial system, and hence recommends its employment in connection with blood-letting in inflammatory affections. Dr. Rognetta adds, that he himself employs, with decided advantage, the salts of quinine in acute ophthalmia, hyposthenic and congestive amaurosis, in gastritis, and in dropsy, symptomatic of abdominal phlogosis. In a series of experiments lately introduced by Dr. Giacomini, he found that the solution of the sulphate of quinine administered in large doses, determined a genuine hyposthenic intoxication, which was only dissipated by the use of mercury, opium, canella, &c. Dr. Rognetta thinks with the Italian physicians, that the limits of tolerance should not be exceeded, and that beyond this a species of poisoning may be induced, known by deafness, blindness, hallucinations, hæmaturia, &c. Dr. Rognetta in conclusion thus resumes his letter:—

1. That the method in question belongs to the Rasorian school.
2. That this method proves the hyposthenic action of the salts of quinine.
3. That we ought not, in the use of this method, to exceed the limits of tolerance.
4. That we can advantageously combine with it the practice recommended by Dr. Bouillaud, (bleeding, *coup sur coup.*)
5. That the combination of the preparations of opium with those of quinine are contra-indicated.

Five severe accidents have lately resulted from this practice, of which two have terminated fatally. Two of these cases occurred at the Hôpital Cochin, and one at La Charité. One patient died immediately after swallowing, at a single dose, *seventy-six and a half grains* (5 grammes) of the salt. At the Hôpital Cochin, a woman labouring under chronic rheumatism, or a disease so called, succumbed soon after the administration of a large dose of the sulphate. A young girl, after the use of the same medicine, became affected with amaurosis, which has already existed for three weeks, in spite of the most appropriate and energetic treatment. The patient at La Charité experienced, at first, pain in the head, then tinnitus, and general agitation, and finally violent delirium, terminating in coma. From this condition he recovered only by the employment of the most active and violent remedies, and after all hope of safety had been abandoned. Except some grave complication interferes with its ordinary termination, acute rheumatism, we all know, is rarely fatal. When death occurs, it is from a phlegmasia of some serous or fibro-serous tissue, and more particularly those of the heart. How then does it happen, that just at the moment these huge doses of the sulphate of quinine become fashionable, acute rheumatism should become so fatal a disease? Is it a singular and inexplicable coincidence? What are the symptoms which precede death in these cases? The usual ones which follows the exhibition of over doses of this medicine, and none other. Is not a demand for future observations a demand for fresh victims? Are there not simpler and surer means of discovering the cause of this extraordinary mortality? Should not humanity and reason dictate that we should suspend this new treatment, and then see if the mortality persists. It has been urged that for a long time Dr. Piorry, of La Pitié, has been in the habit of administering quinine in enormous doses—from a dram to a dram and a half—and without any unpleasant consequences. There is, in the first place, no analogy in the two cases. Dr. Piorry's cases were all intermittent fevers. Does it follow that because a remedy is useful in one disease, it must be so in all others? Besides, Dr. Piorry employs also the neutral salt; whereas that given now is the acid sulphate, which is far more soluble, and probably more is absorbed of it. Moreover, Dr. Piorry now asserts that he obtains the same results from doses of fifteen grains, as he did formerly from those of seventy and ninety grains.

Dr. Serres has just read a very elaborate and favorable report on Mr. Nasmyth's Memoir on the Structure of the Teeth.

A spirit of hyper-therapeutics seems to be becoming fashionable, and if one hospital physician announces as his dose *ten* grains of opium, a *confrère*, unwilling to be outdone, in the next day's journal informs the public that he always gives *twelve* grains, and with invariable success. The surgeons, not to be outdone by the physicians, are pursuing pretty much the same plan with regard to tenotomy. This subject, however, has lately been taken hold of seriously by the Academy of Medicine, and I propose to give you a sketch of their proceedings in relation to it, when concluded. It has occupied the Academy since October, and has excited a great deal of interest. There has been a good deal of sharp discussion between MM. Guérin, Bouvier, Velpeau, Gerdy, &c.

